# Supplementary material for: Generative adversarial reduced order modelling
Source: Sci Rep. 2024 Feb 15;14:3826. doi: 10.1038/s41598-024-54067-z (PMC10869836; doi:10.1038/s41598-024-54067-z)
Supplement: Supplementary file 1 — Supplementary Information. [file 41598_2024_54067_MOESM1_ESM.pdf]

Supplementary Information

1 Hyperparameter tuning

1.1 Hyperparameter tuning baseline models

We report the model details to reproduce the results presented in Table 1. The optimization is carried out using RayTune, searching by grid-search the number of layers and their sizes across 100 different models for each neural network and each experimental problem. For POD-NN, AE-NN, AE-RBF, we fixed the latent dimension to four for the Gaussian test, and sixteen for the Graetz and Lid Cavity problem. In this section, N_u indicates the field solution vector dimension and N_l is the latent dimension size. The activation functions were chosen from a collection of four different activations (ReLU, SiLU, Softplus, Tanh). The Autoencoder is built by choosing the number of layers L, and the output dimension of each layer is chosen by dividing (multiplying) each encoding (decoding) layer input dimension by a chosen F. In particular we choose $L \sim U(1,5)$, and $F \sim U(2,5)$. For the Neural Network in POD-NN we built a neural network by choosing the number of layers L and the layer size S. In particular, we choose $L \sim U(1,8)$, and $F \sim U(5,50)$. For DeepONet we built a neural network by choosing the number of layers L and the layer size S. In particular, we choose $L \sim U(1,4)$, and $F \sim U(20,120)$. For NOMAD we use the same hyperparameter optimization as DeepONet since only the aggregation mechanism changes. Finally, no hyperparameter optimization was performed on (r-)cGAN since only the loss are changing with respect to (r-)GAROM, for which we have done hyperparameter optimization (see 1.2).

POD-NN

POD is performed by truncated singular value decomposition, with rank equals to the latent dimension size. For the Gaussian test case, the neural network is composed 6 fully connected layers of size 43 and ReLU activation function. For the Graetz test case, the neural network is composed of 2 fully connected layers of size 24 and 64 with ReLU activation function. For the Lid Cavity test case, the neural network is composed 6 fully connected layers of size 23 and SiLU activation function.

AE-NN

For the Gaussian test case, the neural network is composed 5 fully connected layers of size 45 and Tanh activation function. The autoencoder comprises five layers of size $[N_u/2, N_u/4, N_l, N_u/4, N_u/2]$ with Tanh activation. For the Graetz test case, the neural network is composed of 2 fully connected layers of size 24 and 64 with ReLU activation function. The autoencoder comprises six layers of size $[N_u/3, N_u/6, N_l, N_u/6, N_u/3]$ with ReLU activation. For the Lid test case, the neural network is composed 3 fully connected layers of size 35 and ReLU activation function. The autoencoder is composed of five layers of size $[N_u/4, N_u/16, N_l, N_u/16, N_u/4]$ with ReLU activation.

AE-RBF

The Radial Basis Function kernel is Gaussian with leght scale equals to one. For the Gaussian test case, the autoencoder is composed of two layers of size $N_u/2$ and $N_u/4$ with Tanh activation. The autoencoder is composed of five layers of size $[N_u/3, N_u/6, N_l, N_u/6, N_u/3$ with ReLU activation. For the Lid test case, the autoencoder comprises two layers of size $N_u/4$ and $N_u/16$ with ReLU activation.

DeepONet

The DeepONet consists of two sub-networks, one for encoding the parameters defining the differential problem (branch network), and another for encoding the locations for the output functions (trunk network). We choose the same architectures for the hidden layers of trunk and branch networks. For the Gaussian test case, the networks are composed of two layers of size 115 with Tanh activation. For the Graetz test case, the networks are composed of three layers of size 104 with Tanh activation. For the Lid Cavity test case, the networks are composed of one layer of size 99 with Tanh activation.

NOMAD

The NOMAD network is the same as the DeepONet network with the only difference in how the branch and trunk networks are organised. Specifically, following the same procedure as the one indicated in the original article[1], the branch network takes a parameter and outputs its embedding. This is concatenated with the spatial location where the output function is evaluated, and this concatenation is passed to the trunk network.

(r-)cGAN

The conditional GAN and its regularized counterpart are composed of a generator and discriminator network. To have a fair comparison with GAROM, we use the same Generator Network as in GAROM (see Table SI.3), fixing only the noise dimension

to 12 since we found it beneficial for the training. Equally, the (r-)cGAN discriminator network is the same as the encoder of the Discriminator network (fixing encoding dimension $N_l = 120$). Once the encoded variable is concatenated with the conditioning variable, a linear layer is applied, which follows a sigmoid activation to ensure the binary output. Only for the Lid Cavity test the training was done without batching since better performance in terms of training and test error was achieved.

1.2 Hyperparameter tuning (r-)GAROM

We report the model details to reproduce the numerics in Table 1 in *Results*. Let the generator activation function be σ_G, the discriminator activation function be σ_D and N_c^{in}, N_c^{in}, N_c^{out} the hyperparameter to optimize. The general (r-)GAROM architecture is reported in Table SI.1 The optimization is carried out using RayTune, searching by grid-search the number

Table SI.1. GAROM model architecture. The table reports the type of layer, its weights and the activation after each layer. The input dimension is indicated as N_u, noise dimension N_z, conditioning dimension N_c, and latent dimension N_l.

Layer	Generator		Discriminator		Generator Conditioning-net		Discriminator Conditioning-net	
	weights	activation	weights	activation	weights	activation	weights	activation
Fully-connected	$[N_z + N_f, N_u/6]$	σ_G	$[N_u, N_u/3]$	σ_D	$[N_c, N_c^{in}]$	σ_G	$[N_c, N_l/2]$	σ_D
Fully-connected	$[N_u/6, N_u/3]$	σ_G	$[N_c^{in}, N_c^{in}]$	σ_D	$[N_c^{in}, N_c^{out}]$	Identity	$[N_l/2, N_l]$	Identity
Fully-connected	$[N_u/3, N_u]$	Identity	$[N_u/6, N_l]$	σ_D	-	-	-	-
Fully-connected	-	-	$[2N_l, N_u/6]$	σ_D	-	-	-	-
Fully-connected	-	-	$[N_u/6, N_u/3]$	σ_D	-	-	-	-
Fully-connected	-	-	$[N_u/3, N_u]$	Identity	-	-	-	-

of layers and their sizes across 100 different models for each neural network and each experimental problem. The activation functions were chosen from a collection of four different activations (ReLU, SiLU, Softplus, Tanh), and the layer sizes as well as the number of layers were chosen accordingly to a random uniform integer distribution. In order to make an ease comparison on convergence and generalization for the test in the section *Generalization*, and *Robustness* we use the same activation functions for discriminator (ReLU) and generator (SiLU) across all tests. Furthermore, only for those two sections, we set $N_c^{out} = 5N_z$, $N_c^{in} = 2N_z$, $N_c^1 = N_z/4$. For SI.1 we report the best architecture we found, so to make a fair comparison with the best architectures for the baseline models. The details for r-GAROM and GAROM for the different test cases are shown in Table SI.2 and Table SI.3 respectively.

Table SI.2. r-GAROM hyperparameters. The table reports the r-GAROM hyperparameters used in the tests in *Results*.

	Gaussian	Graetz	Lid Cavity
σ_G	SiLU	ReLU	ReLU
σ_D	Tanh	Tanh	ReLU
N_c^{in}	17	31	29
N_c^{out}	35	63	58

Table SI.3. GAROM hyperparameters. The table reports the GAROM hyperparameters used in the tests in *Results*.

	Gaussian	Graetz	Lid Cavity
σ_G	SiLU	SiLU	SiLU
σ_D	Tanh	ReLU	ReLU
N_c^{in}	17	24	24
N_c^{out}	35	60	60

2 Baseline Models training details

In AE-NN, AE-RBF the autoencoder is trained for 1000 epochs, while in AE-NN and POD-NN the artificial neural network is trained for 20000 epochs. DeepONet and NOMAD baselines are both trained for 1000 epochs. The (r-)cGAN model is trained

for 20000 epochs for the Gaussian and Graetz test case, while 2500 epochs were enough to ensure a stable convergence for the Lid Cavity experiment. All the baseline neural networks-based models are optimized with Adam[2] using 0.001 learning rate, minimizing the mean square error loss, and training until stable convergence is evidenced using a batch size of 4, otherwise not stated.

References

1. Seidman, J., Kissas, G., Perdikaris, P. & Pappas, G. J. Nomad: Nonlinear manifold decoders for operator learning. *Adv. Neural Inf. Process. Syst.* **35**, 5601–5613 (2022).

2. Kingma, D. P. & Ba, J. Adam: A method for stochastic optimization. *arXiv preprint arXiv:1412.6980* (2014).
